# Medical Society of the County of Albany, Annual Meeting, November 8th, 1870

**Published:** 1870-11

**Authors:** James S. Bailey


### ART. IV.—*Medical Society of the County of Albany, Annual Meeting, November 8th*, 1870.

REPORTED BY JAMES S. BAILEY, M. D.

The Society met at the City Hall, on the second Tuesday in November, at 3 o'clock p. m.

There was an average attendance, over fifty members being present.

Dr. William H. Craig, president, in the chair.

The minutes of the Semi-Annual Meeting were read and approved.

The chairman of the Censors, Dr. Chas. A. Robertson, reported favorably upon the following named physicians for members:

DR. WILLIAM GEOGHEGAN.
" JAMES D. FEATHERSTONAUGH.
" WILLIAM D. WHEELER.
" CHAD. F. SCATTERGOOD.
" WM. H. F. REYNOLDS.

They were accordingly elected members.

The treasurer, Dr. Andrew Wilson, then read his report, which was accepted.

The president, Dr. Wm. H. Craig, delivered the following annual address:—

GENTLEMEN,

I crave your indulgence while I endeavor to fulfil the task imposed upon me by the By-laws.

A community forms its opinion of a profession, first by its usefulness, and second by the character of its representatives.

The legal profession is judged by the equity of the law, the fidelity of its advocates, and the probity of the judiciary.

The religions of the day are estimated by the morals of the people, and the zeal and faithfulness of the clergy. So with the profession of medicine; it derives its status from the skill of the physician, and the benefits it confers in promoting the health of a community. And thus the standing of our profession is constantly before the public, and necessitates an interest in its progress, which is generally admitted to have equalled, if not excelled, the other modern sciences. As examples of this may be adduced, the marvellous changes which modern pathology has made in our knowledge of the nature and cause of many diseases, together with the valuable contributions which have been made by the various special departments of medicine. The activity with which modern operative surgery has undertaken and rendered successful operations, which a few years ago were counted unjustifiable is worthy of all praise, yet, notwithstanding all the evidence we have of the great strides this science has made in alleviating human suffering, is there not indication of a great want of confidence in the regular profession of medicine, as manifested by the increasing demand for patent nostrums, and the popularity of the irregular kinds of practice.

Now, is it not fair and just for every physician to ask himself what are the causes of such want of confidence, and what should be the remedy. Is not the profession partly responsible for this condition of its science. Are there not obstacles to its advancement which it lies within the power of its followers to remove? A reference to the past history of medicine will show that it has always been characterized by extremes, one system of speculative ideas following another in rapid succession. The past two thousand years have advanced such widely varying theories as Naturalism, Empiricism, Electicism, Stimulism, Vitalism, Chemicism, &c. Coming down to the cellular pathology of our own times, which, together with therapeutics, undoubtedly forms the true principles of modern medical practice, the modern theory of investigating diseases, now in vogue for about half a century, and for the last twenty-five years occupying almost exclusively the mind of the great men in the profession, appears the true system of tracing disease through its organic changes and causes, from the healthy to the diseased condition. The energy with which this modern system of pathology has pushed its investigations, aided by the microscope, sphygmograph, thermometer, etc., together with the books, general and special on the subjects, with societies and associations, reports and journals, is worthy of all praise. It has been the theme of many glowing encomiums, and I would not say a word to detract from the great benefit it has conferred on science in the treatment of many diseases, as well as the aid it affords to a correct diagnosis. Yet I am disposed to say that it has not accomplished what its advocates had hoped for, in alleviating and eradicating those diseases generally considered incurable.

What interest is it to the victim of disease to know that the physician is able, by means of physical signs, to tell him the organic changes which have taken place in the lungs in phthisis pulmonalis, or by microscopical or chemical examination, that he has scirrhus, or Bright's disease, unless he is able to point the means of relief, or by some treatment to prevent or eradicate those diseases which are the great enemies of humanity, numbering their victims yearly by thousands?

Does not this indicate that medicine has been more theoretical than practical in our time. Is it not a little remarkable that just in proportion as this theory has been promulgated, men of high reputation in the profession have lost confidence in therapeutics and therapeutic agents, and rely mostly on nature and hygiene, which were the prevailing ideas of the treatment of disease in the days of Hippocrates, and have had such brilliant advocates recently as Sir John Forbes and Dr. Bigelow. The useless administration of medicines, in many cases, together with the diversity of opinion among physicians regarding the treatment of some diseases, may have caused a partial prejudice in this direction. Thus, in pneumonia, where the conditions are similar, one physician will follow the stimulating treatment, another the antiphlogistic, while still another will rely entirely on nature. Does not this contrariety of opinion serve to destroy the confidence of the public in the regular practice, and induce many to adopt the popularisms of the day.

While these may be some of the reasons that effect the status of the profession, yet I apprehend that the great fault has been in the tendency to advance medicine as a science rather than to make progress in it as an art. To day, while the science of physiology and pathology is constantly undergoing investigation, so that the works on these subjects have to be constantly revised, to keep up with the changes and new discoveries, yet in therapeutics we are satisfied with the old and absurd nomenctature of Cullen, such as lithor-thyptics, deobstruents, &c.

Is it not remarkable how little real persistent scientific effort there has been made to determine the therapeutic effect of agents within the past thirty years, while the pharmaceutist and chemist have performed such laudable work in the discovery of new remedies, and in furnishing pure and reliable articles of medicine. I except in these reflections the scientific efforts made by the late Dr. Tulley, whose investigations gave promise of important contributions to the Materia Medica. In view of the great light which physiology has thrown on the nervous system within the past few years, would it not stimulate to more investigation in this direction. Is not modern chemistry becoming every day synthetical as well as analytical. May we not reasonably look in this channel for the means of counteracting the horrors of hydrophobia, penetrating the mysteries of Bright's disease. and checking the fearful fatality of phthisis.

Are not the elements susceptible of accomplishing this work all around us in nature, and simply awaiting the genius of the "coming man" to discover them.

A few years since, when the scientific naturalist supposed their work about completed on the subject of the *fennue genera*, Prof. Agassiz made his celebrated visit to the great River Amazon, where, in a few months, he discovered more than three times the number of species of fish than had been known before. So I believe the therapeutic department of medicine will be yet illuminated by like patient and bold research.

If a comparison were permitted of the relative benefits to the human race, what discovery has modern pathology made to equal the utility of anæsthesia. Yet twenty-five years ago the man that had asserted as possible, that which has been accomplished would have been laughed at as a madman.

I believe that we are on the eve of a great re-action in these departments of our profession, and that the next *decade* will develop many marvellous changes in our knowledge of therapeutics, in which practical medicine will become more demonstrative and less theoretical than heretofore.

The recent experiments made by Dr. Hughes Bennet and others on *animals* prove almost conclusively that mercury does not increase the biliary secretions; and yet this specific effect has been accorded to this agent more confidently than to any other article of the Materia Medica.

Without espousing the conclusions of these eminent scientists, it seems proper to assent that what the times, and the enlargement of our professsonal knowledge demands, is a more systematic and scientific effort to determine the true therapeutical relation of certain medicinal agents, with reference to modern discoveries in physiology and pathology; or in the language of another, "what we require is not unfounded assertion and vague speculation, but positive knowledge, something that will contribute to the science of therapeutics a more certain and less conjectural character."

I approach the second part of my subject with some hesitation, for it becomes necessary in defining the character of some of the representations of our profession to animadvert on certain practices that tend to lower them in public confidence, and exposes them at times to seemingly merited censure, while the modern practitioner does not parade, as in former times, such fantastic *insignia* of his office, as the powdered wig and gold headed cane; nor affect a profound mystery while administering simple rhubarb pills or magnesia powders; yet there are those who bid for public notice as the especial champions of some popular remedy, such as cod liver oil or carbolic acid, or the hydrate of chloral, and laud these as the universal panacea for the cure of "the ills that flesh is heir to."

Can we refrain from condemning the inexperienced specialist, who, upon the mere suspicion of metritis, or other uterine disorder, rushes to the use of the speculum in wanton disregard of the most delicate sensibilities, a procedure which the honorable physician will only resort to when a correct diagnosis imperatively demands it. Or have we not reason to lament the too prevailing practice of professional experts appearing as medico-legal witnesses in courts of justice, who, for a valuable consideration, will discover a convenient *frenzy* as a justification to shield a criminal, and thwart justice.

And if you will permit a single word of criticism more, have we not sometimes reason to fear that the advertising in the public prints of cases treated, and of skillful surgical operations performed, may be deemed derogatory to professional dignity and honor.

Gentlemen, I would not have you think that I have no admiration for our profession because I have made mention of some of the causes which in my opinion impairs it in the public confidence, I still believe it noble in all its efforts to alleviate human suffering.

And while it may have its dark spots, like the great luminary of the universe, yet, like the sun, it warms and vivifies nature into bloom, health and beauty.

That modern medicine by its science of hygiene is preventing disease and is promoting the longevity of the human race, is entitled to more confidence and esteem than is commonly awarded to it. Only those persons who have lived in foreign countries, as in China or Japan, can rightly estimate or appreciate this truth.

The assertion which we frequently hear that medicine is not one of the exact sciences, is not to be wondered at when we contemplate the vastness and magnificence of the laws that govern the human body, as well as the minutiæ of the cells that constitute its tissues, for modern chemistry too, might exhaust the Greek alphabet for numerals to express the variety of substances which can be formed. "Berthelot makes a calculation of the number of combinations with acids of certain alcohols. He says, if you gave each a name, allowing a line for the name, then print 100 lines in a page, and make volumes of 1,000 pages, and place a million of volumes in a library. you would need 14,000 libraries for your catalogue."

Surely such a science might be said to approach the infinite, and to be above the comprehension of finite man, and demand the formation of special departments of study, these to be multiplied by the thousand, and then after patient years of investigation, comprehend all that may be evolved from so illimitable a field.

Does it not then become the representatives of a profession, with such a career of discovery and usefulness before it, to discard all effete and charlatan practices. And then may we not hope to elevate the profession of medicine so, that it may stand before the world like a temple illuminated in every part, and not resemble a house which reflects light from only a single apartment.

In closing allow me to congratulate you upon the interest and zeal which have been manifested in our society during the past year. Besides the Semi-Annual Meeting, there have been held other meetings in the evening, usually at intervals of two weeks, the average attendance at which was 29. Although so frequently occuring, a decided interest was manifested in them from first to last; this interest was shown not only by the numbers present, but also by the numerous reports, and the great variety of subjects that were discussed relating to the different departments of medicine and surgery, as well as to the medical ethics. In the number and character of the reports of medical and surgical cases there has been a decided improvement over any previous year. Indeed during these meetings between 70 and 80 cases have been presented, most of them in detail, and accompanied with pathological specimens. Many of these reports were the occasion of useful and interesting discussion.

I have still a painful duty to perform. All are not here to-day who were with us a year ago. We miss the familiar face of our associate, Dr. Alfred Wands, of Cohoes, an old and esteemed member of this society. Although I had known Dr. Wands for several years, yet my relations with him were not as intimate as many of our brethren of Cohoes, where he spent most of his professional life, and succeeded in building up a large practice, and enjoyed the confidence and esteem of all who knew him. His death, in the prime of life, is the cause of sincere mourning, and has severed ties, to family, profession and community, which can never be restored.

At the conclusion of which, Dr. Levi Moore moved that the thanks of the Society be presented to Dr. Craig, for his able address, and that he be requested to furnish a copy, to be forwarded to the State Medical Society, for publication in its transactions.

It was adopted unanimously.

The balloting was then proceeded with, and the following named gentlemen were elected to the offices mentioned:—

DR. WM. H. BAILEY, President.
" ANDREW WILSON, Vice-President.
" C. H. PORTER, Secretary.
" D. V. O'LEARY, Treasurer.

DR. MILTON M. LAMB,

" J. R. BOULWIN,

" STAATS WINNE,

" J. P. WHITLERETE,

" JNO. F. CROUNSE,

} Censors.

DR. AMOS FOWLER,

" HENRY MARCH,

" JOHN FERGUSON,

" JOSEPH LEWI,

} Delegates to American Medical Association.

The following gentlemen were proposed for membership:—

DR. W. H. MURRAY,
" LORENZO HALL,
" J. H. FRENCH.

Dr. A. A. Edmeston offered his resignation to the Society as delegate of the N. Y. State Medical Society, stating that his health was such as to render him unable to properly represent the County Society in that body.—Accepted.

Dr. John P. Whitlete was nominated and elected to fill the unexpired term, made vacant by Dr. Edmeston's resignation.

Dr. D. T. Crothers read an interesting paper on the therapeutic power of oxygen gas.*

*A synopsis of this paper will be published in our next number.

